# Small striatal huntingtin inclusions in patients with motor neuron disease with reduced penetrance and intermediate HTT gene expansions

**DOI:** 10.1093/hmg/ddae137

**Published:** 2024-09-13

**Authors:** Anna-Karin Roos, Erica Stenvall, Emmy Skelton Kockum, Kornelia Åman Grönlund, Helena Alstermark, Anna Wuolikainen, Peter M Andersen, Angelica Nordin, Karin M E Forsberg

**Affiliations:** Department of Clinical Sciences, Neurosciences, Umeå University, Norrlands University Hospital, Building 6 M, Floor 4, Umeå SE-90184, Sweden; Department of Medical Biosciences, Umeå University, Norrlands University Hospital, Building 6 M, Floor 2, Umeå SE-90184, Sweden; Department of Medical Biosciences, Umeå University, Norrlands University Hospital, Building 6 M, Floor 2, Umeå SE-90184, Sweden; Department of Clinical Sciences, Neurosciences, Umeå University, Norrlands University Hospital, Building 6 M, Floor 4, Umeå SE-90184, Sweden; Department of Clinical Sciences, Neurosciences, Umeå University, Norrlands University Hospital, Building 6 M, Floor 4, Umeå SE-90184, Sweden; Department of Medical Sciences, Neurology, Uppsala University, Uppsala University Hospital, Entrance 85, Floor 2, Uppsala SE-75185, Sweden; Department of Clinical Sciences, Neurosciences, Umeå University, Norrlands University Hospital, Building 6 M, Floor 4, Umeå SE-90184, Sweden; Department of Medical Biosciences, Umeå University, Norrlands University Hospital, Building 6 M, Floor 2, Umeå SE-90184, Sweden; Department of Clinical Sciences, Neurosciences, Umeå University, Norrlands University Hospital, Building 6 M, Floor 4, Umeå SE-90184, Sweden

**Keywords:** Amyotrophic lateral sclerosis, *C9ORF72HRE*, huntingtin inclusions, somatic mosaicism

## Abstract

Short tandem repeat expansions in the human genome are overrepresented in a variety of neurological disorders. It was recently shown that huntingtin *(HTT)* repeat expansions with full penetrance, i.e. 40 or more CAG repeats, which normally cause Huntington’s disease (HD), are overrepresented in patients with amyotrophic lateral sclerosis (ALS). Whether patients carrying *HTT* repeat expansions with reduced penetrance, (36–39 CAG repeats), or alleles with intermediate penetrance, (27–35 CAG repeats), have an increased risk of ALS has not yet been investigated. Here, we examined the role of *HTT* repeat expansions in a motor neuron disease (MND) cohort, searched for expanded *HTT* alleles, and investigated correlations with phenotype and neuropathology. MND patients harboring *C9ORF72* hexanucleotide repeat expansions (HREs) were included, to investigate whether *HTT repeat expansions* were more common in this group*.* We found a high prevalence of intermediate (range 5.63%–6.61%) and reduced penetrance (range 0.57%–0.66%) *HTT* gene expansions in this cohort compared to other populations of European ancestry, but no differences between the MND cohort and the control cohort were observed, regardless of *C9ORF72HRE* status. Upon autopsy of three patients with intermediate or reduced penetrance *HTT* alleles, huntingtin inclusions were observed in the caudate nucleus and frontal lobe, but no significant somatic mosaicism was detected in different parts of the nervous system. Thus, we demonstrate, for the first time, huntingtin inclusions in individuals with MND and intermediate and reduced penetrance *HTT* repeat expansions but more clinicopathological investigations are needed to further understand the impact of *HTT* gene expansion-related pleiotropy.

## Introduction

The nervous system is vulnerable to gene expansions, and pathological repeat sequences can cause a variety of neurological diseases. Amyotrophic lateral sclerosis (ALS) is a heterogeneous neurodegenerative syndrome that primarily affects the motor neurons of the brain, brainstem and spinal cord [1]. At least 20%–26% of ALS patients have a hereditary predisposition [2], and mutations in more than 40 genes have been implicated in ALS development, most often as a dominant Mendelian trait with varying degrees of penetrance. In populations with European ancestry, a pathogenic hexanucleotide repeat expansion (HRE) in *C9ORF72,* is the most commonly identified genetic cause of ALS [3, 4]. Interestingly, Huntington’s disease (HD) and ALS have been reported to coexist in the same individuals [5, 6] or in the same families [7]. A recent study revealed that repeat expansions with the full penetrance length (40 or more CAG repeats) in *HTT*, which normally causes HD, are overrepresented in ALS patients, and they observed huntingtin aggregates in the striatum and frontal cortex in the CNS of these individuals [8].

Less is known about the risk of ALS caused by *HTT* alleles with fewer repeats. Expansions with 36–39 repeats cause HD with reduced penetrance (RP), and alleles with 27–35 repeats (intermediate alleles, IA) usually do not cause HD. Reports of individuals with *HTT* IAs, who nonetheless become symptomatic, might be explained by a predisposition for somatic expansion, which causes higher repeat numbers in vulnerable tissues [9]. A higher degree of somatic expansion in HD patients is linked to variants in DNA maintenance and repair genes [10–12], resulting in worse outcomes of HD [13].

The population in northern Sweden has a high prevalence of *HTT* IA (6.8%) [14], similar to other populations with European ancestry (5,2%-6,3%) [15–17]. Individuals with *HTT* IA have an increased risk of depression [18] and behavioral abnormalities [19] compared to individuals with lower numbers of *HTT* repeats. Here, we compared the prevalence of *HTT* IA and RP expanded alleles in a Swedish cohort with motor neuron disease (MND). We also included individuals with *C9ORF72HRE,* to investigate whether *HTT* expansions were more common in this group, thus revealing possible underlying DNA repair dysfunction. Three of the patients had undergone autopsy, and we searched for *HTT-*related pathology and measured the grade of somatic expansion in different tissues. We found small striatal huntingtin inclusions in all three individuals carrying IA and RP *HTT* alleles. To our knowledge, this is the first study to investigate histopathological huntingtin inclusions in MND patients carrying *HTT* IA and RP expanded alleles.

## Results

### There was a high prevalence of *HTT* IA and RP expanded alleles, but no significant differences were found between the MND cohorts and nonneurological control group

The prevalence of gene expansions in the MND cohort with and without *C9ORF72HRE* were 5.63% and 6.61%, respectively, for *HTT* IA and 0.66% and 0.58%, respectively, for *HTT* RP. The corresponding percentages in the control group were 5.97% and 0.57%, respectively (Table 1). No statistically significant difference was detected in the prevalence between the two MND groups or the control group (Fisher’s exact test p value of 0.983). However, the overall prevalence of IA and RP *HTT* alleles was high in both the MND cohort and control group, as in other general populations with European ancestry [15–17]. All individuals with RP *HTT* alleles in the MND cohort and control group were descendants of families from northern Sweden.

**Table 1:** Baseline characteristics of patients in the MND cohorts and control group and prevalence of expanded HTT alleles in each group.

	**MND with *C9ORF72HRE***	**MND without *C9ORF72HRE***	**Control group**
Number of individuals	302	514	352
Median age of onset (years)	58.7 (range 31–80)	60.3 (range 20–93)	age at sampling event 62.6 (range 27–89)
Percentage women	50.7%	41.6%	53.7%
Mean *HTT* CAG repeat number	18.5 (range 9–38)	18.4 (range 9–40)	18.6
Median *HTT* CAG repeat number	17	17	17
Individuals with full penetrance *HTT* gene expansion (40 or more repeats) (%)	0	1 (0.19%)	1 (0.28%)
Individuals with reduced penetrance *HTT* gene expansion (36–39 repeats) (%)	2 (0.66%)	3 (0.58%)	2 (0.57%)
Individuals with intermediate *HTT* gene expansion (27–35 repeats) (%)	17 (5.63%)	34 (6.61%)	21 (5.97%)

Information about age of onset is missing for 36 individuals in the C9ORF72HRE group and 78 individuals in the group without C9ORF72HRE. C9ORF72HRE, hexanucleotide repeat expansion in the C9ORF72 gene; HTT gene, gene associated with Huntington’s disease.

We identified one individual with full penetrance *HTT* gene expansion in the control group (352 (0.28%)) and one in the MND cohort (816 (0.12%)). The individual with *HTT* expansion (*HTT* 40/18) in the MND group had no family history for dementia, neurological- or psychiatric disease. At the age of 75, she developed classical symptoms of progressive upper and lower motor neuron disease (bulbar paresis and emotional lability) with typical EMG and plasma NfL findings. She died from respiratory failure 46 months after the first paretic symptom. Other than early vertical gaze palsy, no atypical ALS- or HD-related signs or symptoms were observed during the disease course.

### Intermediate *HTT* gene expansions do not influence the motor neuron disease phenotype

Clinical data for individuals with *HTT* IA alleles are summarized in Table 2 and Supplementary Table 6, data for individuals with *HTT* RP alleles are summarized in Supplementary Table 7. No significant differences in phenotype were observed between patients with or without *HTT* IA. The concomitant presence of *C9ORF72HRE* did not change this interpretation (p values of 0.847 and 0.332, respectively). The number of individuals with RP *HTT* expansions was small, but interestingly, although extrapyramidal symptoms were seldom described, two of the patients with RP *HTT* without concomitant *C9ORF72HRE* showed extrapyramidal features during the disease course, in addition to classical MND symptoms (Supplementary Table 7).

**Table 2:** Clinical characteristics of individuals in the MND cohorts.

	**Individuals with *C9ORF72HRE***		**Individuals without *C9ORF72HRE***	
	**Without *HTT* IA**	**With *HTT* IA**	**Without *HTT* IA**	**With *HTT* IA**
Numbers	283	17	476	34
Percentage women (%)	50,9	41,2	40.1	64.7
Median age of onset (years)	61.1	58.2	63.8	61.1
Median disease duration in years (mean)	2.4 (3.1)	2.8 (3.2)	3.0 (4.2)	3.0 (3.1)
Spinal phenotype (%)	55.6	66.7	65.6	71.8
Bulbar phenotype (%)	34.7	26.7	31.1	25
Cognitive phenotype (%)	9.3	6.7	1.2	3.1
Respiratory phenotype (%)	0.4	0	2.1	0
Phenotype unknown (numbers)	15	2	49	2

Individuals with reduced penetrance and full penetrance HTT alleles are described separately. C9ORF72HRE, hexanucleotide repeat expansion in the C9ORF72 gene; HTT, gene associated with Huntington’s disease; IA, allele within intermediate range.

### Clinicopathological analysis revealed huntingtin inclusions in caudate nuclei in patients with RP and IA *HTT* alleles

Two of the patients with RP and one patient with *HTT* IA CAG repeat expansions in our cohort underwent autopsy. A clinical summary is listed in Supplementary Table 8. The first patient was a man carrying 36 *HTT* CAG repeats who, at age 71 years, presented with progressive paresis in the left arm and hand and died 4 years later from respiratory failure with generalized ALS disease. In the first half of the course of disease, he showed typical features of a classical ALS. Tremor in the hands was described 14 months after onset of paresis and mild executive dysfunction (ECAS 88/136, cut off for abnormality 107) and episodes of anxiety were first observed at 21 and 33 months, respectively, after onset of paresis. Episodes of dystonia in the right arm were noted 28 months after onset of paresis. Chorea or other extrapyramidal symptoms were not observed.

Postmortem examination revealed macroscopic thinning of the anterior roots of the spinal cord. The brain, including the striatum (Fig. 2a), was regular in size and had no atrophy. Microscopic assessment revealed loss of motor neurons in cervical, thoracic and lumbar spinal cord and in the hypoglossal nuclei. Staining with anti-pTDP-43 antibodies revealed skein-like and granular compact inclusions in motor neurons at all levels of the spinal cord (Fig. 2f) and in the hypoglossal nuclei (Fig. 2e) and, to a lesser extent, in the motor cortex and hippocampus. The same regions also exhibited positive staining for p62. In addition, p62-positive inclusions were observed in the frontal and temporal lobes, basal ganglia, cerebellum and olivary nucleus. Misfolded SOD1 inclusions were present in spinal cord motor neurons and hypoglossal nuclei (Supplementary Fig. 1c). The Alzheimer’s disease NIA-AA ABC score was low (A2B1C1), [20]. No cerebral amyloid angiopathy (CAA) was detected, and no α-synuclein staining was observed. Interestingly, upon staining with two different *HTT* antibodies (Supplementary Table 5), we detected small round immunoreactive inclusions in the caudate nuclei. The inclusions appeared to be extracellularly located (Fig. 2b-d).

The second patient evaluated postmortem was a man carrying 36 CAG repeats in *HTT* who, at age 83, developed dysarthria and dysphagia. He was diagnosed with the progressive bulbar paresis (PBP) variant of ALS and died 15 months later from pneumonia and respiratory insufficiency. He developed short-term memory deficits at the end of his life but showed no signs of extrapyramidal symptoms (Supplementary Table 8, Patient #2). Postmortem examination revealed a regular size of the brain and striatum and thinning of the anterior spinal roots. Microscopically, loss of motor neurons was observed at all levels of the spinal cord (Fig. 3a) as well as in the hypoglossal nuclei. Staining with anti-pTDP-43 antibodies revealed granular and compact skein inclusions in these areas (Fig. 3b). In the motor cortex, only a few small neurons had cytoplasmic inclusions (Fig. 3d). Low levels of pTDP-43 inclusions were also observed in the basal ganglia (Fig. 3c), mesencephalon and hippocampus, which all exhibited positive staining for p62. Moreover, p62 inclusions were observed in the olivary nucleus. Misfolded SOD1 inclusions were present in spinal cord motor neurons and hypoglossal nuclei (Supplementary Fig. 1e). This patient had no pathology indicative of Alzheimer’s disease, the NIA-AA ABC score was A0B2C0. The tauopathy findings indicated primary aging-related tauopathy (PART). Moreover, small vessel disease, including hemosiderin deposits but no lacunar infarcts, was observed. No α-synuclein staining was observed. Interestingly, small round immunoreactive inclusions of huntingtin in the caudate nuclei were also detected in this patient (Fig. 3f). Small p62 inclusions of the same size and shape as the huntingtin inclusions were observed in the same area (Fig. 3e).

The third autopsy case was a 67-year-old woman carrying 33 CAG intermediate repeats in *HTT*, who presented with paresis in the right arm that eventually progressed to include all four limbs and the respiratory muscles. She died 62 months after onset and never showed any clinical signs of upper motor neuron lesions, hence her clinical diagnosis was sporadic progressive muscular atrophy (PMA) (Supplementary Table 8, Patient #3). She had no extrapyramidal signs or symptoms during the course of disease but experienced anxiety and short-term memory loss. Macroscopic examination revealed that the cerebrum and the striatum were regular in size. Microscopically, selective loss of motor neurons was observed at all levels of the spinal cord but to a lesser extent in the lumbar region. Some motor neurons had a swollen eosinophilic appearance (Fig. 4d). Loss of neurons was also observed in the hypoglossus nucleus (Fig. 4a). Somewhat surprisingly, staining with an anti-phospho-TDP-43 antibody was negative without cytoplasmic translocation in the spinal cord (Fig. 4f), medulla oblongata, mesencephalon, hippocampus, and all cortical areas, including the prefrontal and motor cortex, basal ganglia and caudate nucleus. Staining for nonphosphorylated TDP-43 revealed regular nuclear immunoreactivity without cytoplasmic inclusions. Staining for p62 in the motor cortex revealed cytoplasmic inclusion in a single neuron in layer 3, but the results were otherwise negative. Staining for P62 was weakly positive in the hypoglossal and olivary nuclei as well as in the stratum moleculare and granulare of the cerebellum, hippocampus, frontal cortex and parietal cortex. The red nucleus in the mesencephalon had cytoplasmic neuronal inclusions, and threads were observed in the substantia nigra. The basal ganglia had few p62 inclusions. Interestingly, in the cervical spinal cord, p62 staining revealed large, round, diffuse, few skein-like inclusions in the cytoplasm of motor neurons (Fig. 4e). These inclusions stained negative for FUS, Tau (AT8) SOD1 and α-synuclein (Supplementary Fig. 1a, b, d and f). Additionally, small vessel disease, with hemosiderin deposits in the striatum and basal ganglia as well as in all cortical regions, was observed. No lacunar infarctions or CAA were observed. The NIA-AA ABC score was low for Alzheimer’s disease, (A1B1C1) [20]. In summary, motor neuron pathology without typical TDP-43 staining was seen and a whole genome sequencing is undertaken to elucidate the underlying pathology (work in progress). Staining with an antibody against huntingtin revealed a few extracellular huntingtin inclusions in the frontal cortex (Fig. 4b) and pons (Fig. 4c). To confirm the specificity of the findings, we examined the striatum in ten control individuals—three patients with Parkinson’s disease, three patients with ALS and four patients without neurodegenerative disease—using two *HTT* antibodies. All ten patients were negative for huntingtin inclusions (not shown).

### Low-grade somatic instability in autopsied individuals with intermediate or reduced penetrance *HTT* expansions

All three patients were further analyzed to assess *HTT* expansion mosaicism. Both neuronal and peripheral tissues were examined (Supplementary Table 2). Using fragment length analysis, we found no difference in the size of the main *HTT* alleles between neuronal and peripheral tissues. Furthermore, the allele sizes did not differ from the sizes that were measured in DNA extracted from blood leucocytes. We also performed RP-PCR to assess somatic instability. The RP-PCR pattern was similar in all the samples from the three patients (Fig. 5), with signs of low-grade somatic instability in the samples. In Patient #1, somatic instability was slightly more evident in the nucleus caudatus (Fig. 5a) and basal ganglia (data not shown).

## Discussion

By analyzing the prevalence of *HTT* IA and RP expansions in a Swedish MND cohort consisting of patients with or without *C9ORF72HRE* mutations, we confirmed a high prevalence of *HTT* repeat expansion alleles in northern Sweden, as previously shown in non-MND-related studies [14, 21]. The prevalence of RP *HTT* expansions in the control group and the MND group (0.57%–0.66%) was even higher than that reported by Sundblom et al. (0.4%), indicating that *HTT* alleles with higher repeat numbers are common in the general population of northern Sweden [14].

No difference in prevalence between the MND cohort and the control group was detected, regardless of the presence of full penetrance, reduced penetrance or intermediate *HTT* alleles. Thus, our study does not support that *HTT* allele size affect ALS risk. This finding is consistent with previous results from a sporadic ALS cohort [22]. However, in a recent study by Dewan et al. [8], a higher prevalence of full penetrance *HTT* alleles in MND patients than in controls was reported, and inclusions of huntingtin were found in the frontal cortex in two patients carrying full-length mutations. Since full penetrance mutations in the *HTT* gene are rare in patients with ALS, we cannot exclude the possibility that our sample size was too small to observe this difference. The individual in our MND cohort with full penetrance *HTT* expansion presented with typical signs of MND, except for vertical gaze paresis. A prediction model for age of onset and penetrance for HD [23], estimated an 87% probability that she would have motor symptoms caused by HD. Unfortunately, autopsy was not performed, and thus, no information on histopathology or possible somatic expansion was available.

The clinical phenotype resulting from *HTT* gene expansion is heterogeneous and exhibits age-dependent penetrance [24]. In our cohort, no difference was observed in MND phenotype between individuals carrying *HTT* IA gene expansions and those without (Table 2). Extrapyramidal symptoms were rarely reported in medical records, and since this was a retrospective study, underreporting of non-motor neuron symptoms and signs was possible. Our results are supported by a small Italian study that found no significant difference in phenotype between ALS patients with or without *HTT* IA alleles but a tendency toward a higher prevalence of the spinal phenotype in individuals with *HTT* IA [25]. In our study, there were too few individuals with *HTT* RP expansions to draw conclusions. Interestingly, two of the five *HTT* RP individuals exhibited extrapyramidal symptoms (Supplementary Table 7).

The typical neuropathological finding in HD is a variable degree of neuronal loss and gliosis in the striatum, and the earliest changes are observed in the medial paraventricular portions of the caudate nucleus [26]. Immunostaining for ubiquitin in the caudate nucleus of a presymptomatic individual with 37 CAG repeats was reported [27], and the results indicated that *HTT* RP expansions result in histopathological changes regardless of clinical symptoms. In a recent study by Hickman et al. [28], neocortical huntingtin inclusions were found to correlate with CAG repeat numbers; therefore, this study suggested that inclusions could also be formed in individuals carrying intermediate expansions. Our present study confirms this theory and describes, for the first time, huntingtin inclusions in the caudate nucleus and frontal cortex of three MND individuals carrying *HTT* RP and IA gene expansions (Fig. 2b-d, Fig. 3f and Fig. 4b,c), in addition to classical ALS-related pathology (Fig. 2e-f, Fig. 3a-e and Fig. 4a + d-f). A possible toxic effect of these small extracellular huntingtin inclusions can only be hypothesized on. Most likely they are just a result of repeat expansions in the *HTT* gene and do not affect onset of a MND since they are rare and only found in the caudate nucleus and frontal lobe. However, since ALS can be viewed as a multi-step disease process [29] one can speculate that *HTT* repeat expansions and its corresponding huntingtin inclusions, could be one of many risk factors which in combination with other risk factors can lead to onset of a MND. Depending on regional CNS tissue vulnerability and already existing pathology, these inclusions could speculatively contribute to the risk of developing motor neuron disease, and/or influence age of onset, disease progression or even type of neurodegenerative disease. Other neuropathological studies of individuals with *HTT* IA are rare, but polyglutamine inclusions have been reported in two individuals with a diagnosis of MSA [30].

**Figure 1:**
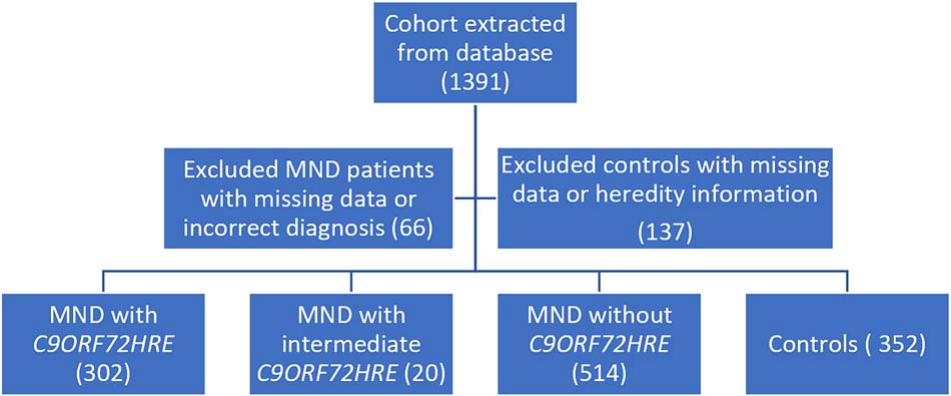
Description of selection of individuals for the MND cohort and control group from the database. MND, motor neuron disease; C9ORF72HRE, hexanucleotide repeat expansion in the C9ORF72 gene.

**Figure 2:**
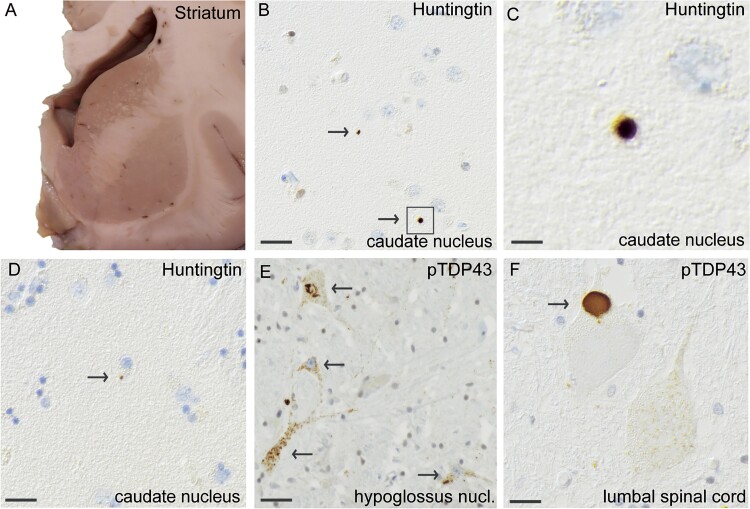
Histopathological findings of huntingtin and pTDP-43 inclusions in MND patient #1, carrying reduced penetrance HTT repeat expansion (HTT 36/19). A coronal section of a fresh brain showing the striatum (A) that has regular size and no atrophy. Staining with an antibody against huntingtin shows small extranuclear huntingtin staining (arrows) in the caudate nucleus (B), enlarged in (C). The area of huntingtin staining was small, and huntingtin was located in the extranuclear space (D). Using the pTDP-43 antibody against phosphorylated TDP-43, cytoplasmic TDP43 inclusions are seen in the motor neurons of nucleus hypoglossus (E) and large granular cytoplasmic pTDP43-inclusions were observed in lumbar spinal cord (F). Scale bar represents 100 μm in b-f.

**Figure 3:**
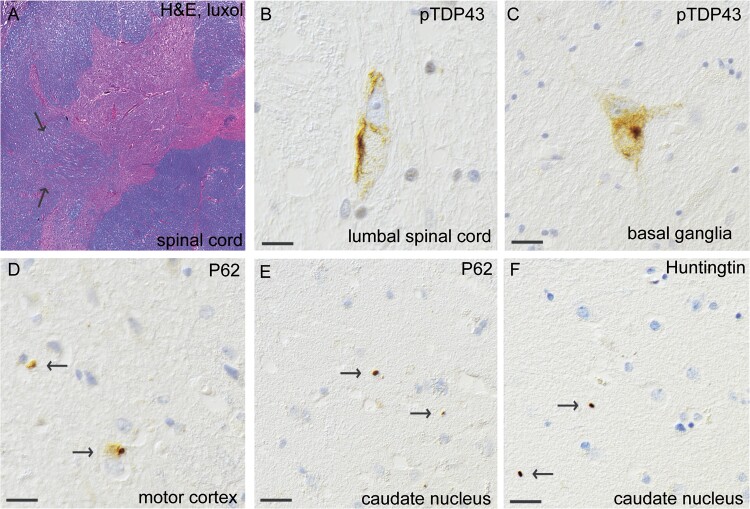
Histopathological findings of inclusions of pTDP-43, p62 and huntingtin in MND patient #2, carrying reduced penetrance HTT repeat expansion (HTT 36/17). (A) Thoracic spinal cord stained with H&E and luxol. Note the myelin pallor (arrows) in the pyramidal tract. Skein-like granular cytoplasmic pTDP43-inclusions were observed in lumbar spinal cord (B) and in basal ganglia (C). On staining with an antibody against P62, granular cytoplasmic inclusions are seen in motor cortex (D). Extranuclear inclusions are seen in nucleus caudatus stained with P62 (e, arrows) and are also seen when stained for huntingtin (f, arrows). Note the similarity of the inclusions. Scale bar represents 100 μm in b-f.

**Figure 4:**
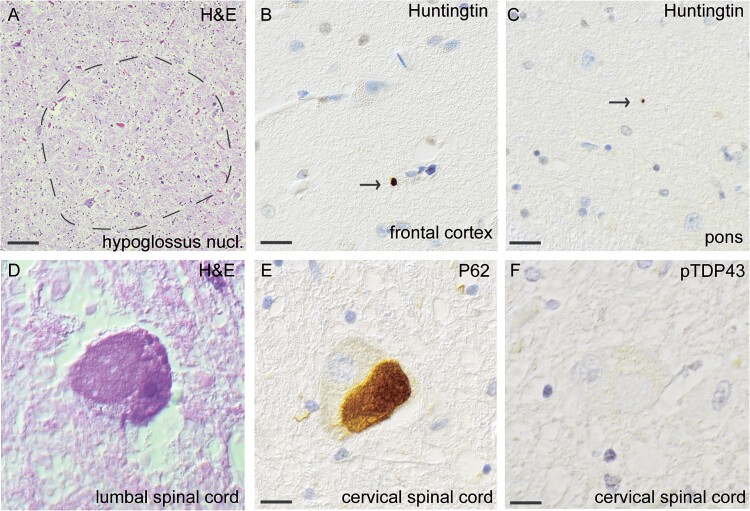
Histopathological findings of huntingtin and p62 inclusions, but no pTDP43-inclusions, in MND patient #3, carrying HTT repeat expansion within intermediate range (HTT 33/22). Loss of neurons in the hypoglossus nucleus stained with H&E (A). Extranuclear small inclusions of huntingtin were seen in the frontal cortex (B) and in pons (C). Swollen eosinophilic motor neuron in lumbar spinal cord stained with H&E (d). Note the swollen eosinophilic appearance. Staining with an antibody against p62, large round p62-positive inclusions are observed in the cervical spinal cord (E). No inclusions are seen in the cervical spinal cord in when stained with pTDP-43 (f). Scale bar represents 20 μm in a, 100 μm in b-f.

**Figure 5:**
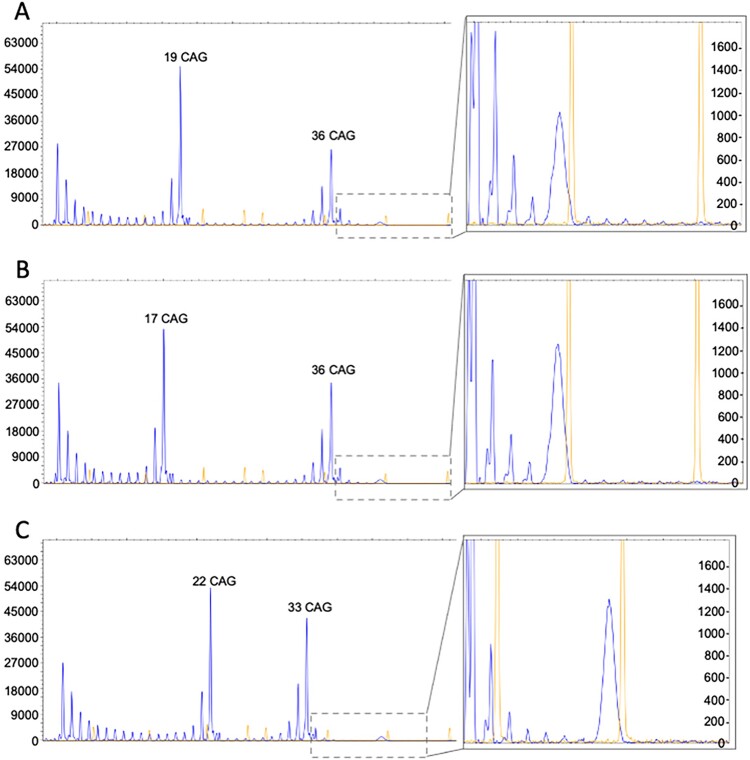
Low-grade somatic instability of HTT repeats in three patients with reduced penetrance or intermediate alleles. RP-PCR was performed to assess CAG-repeat numbers in HTT. (A-C) Repeat numbers in nucleus caudatus of autopsy samples from three individuals. Y-axis indicate signal strength of the analyzed sample. The high and narrow peaks in the enlarged section (to the right in the picture) are size standard peaks used for size assessment.

Somatic instability in repeat expansion diseases results in variable copy numbers in different parts of the CNS [31–33] and complicates the definition of intermediate repeat length, as the true repeat number cannot be fully identified. Interestingly, in our study, RP-PCR analysis revealed low-grade somatic instability in the caudate nucleus and frontal lobes of the individual with *HTT* IA (Fig. 5), which might explain why huntingtin inclusions were found in these regions. Additionally, individual DNA repair ability might influence and modify the grade of somatic instability, but this possibility was not investigated here [12, 34].

One can hypothesize that, individual conditions such as mosaicism, DNA repair ability and the presence of intermediate or reduced penetrance expanded alleles in several genomic loci might lead to a total nucleotide repeat load that exceeds the homeostasis repair machinery in vulnerable motor neurons, resulting in cellular failure and cellular protein aggregation.

There are limitations to the present study. For a few patients, clinical information was incomplete, in particular regarding cognitive, behavioral and/or extrapyramidal symptoms. In later stages of disease, when focus of clinical ALS care is on palliation, respiratory and nutritional support, detailed neurological examinations frequently were missing. Additionally, since only healthy relatives without known genetic relationships with MND patients were recruited as controls, the control group was not age- or sex-matched.

To summarize, we confirm a high prevalence of *HTT* repeat expansion alleles in northern Sweden and demonstrate for the first time the presence of small striatal huntingtin inclusions in addition to typical MND histopathology in three MND patients with *HTT* RP and IA expansions. However, we could not confirm that *HTT* allele size affect ALS risk. More clinicopathological investigations on the MND phenotype and *HTT* gene expansions are needed to further understand the biological mechanism underlying *HTT* gene expansion-related pleiotropy and pathogenesis.

## Materials and methods

### Participants

The study cohort is described in Fig. 1. Patients were diagnosed, according to the revised El-Escorial criteria [35], adhering to the EFNS clinical diagnostic algorithm for ALS [36] and recruited to participate in research at the Umeå University Hospital, Sweden, between 1994 and 2023. The final cohort included 302 patients with *C9ORF72HRE* and 514 patients without *C9ORF72HRE* whose *HTT* gene status was analyzed. We included individuals with classical ALS, progressive bulbar paresis (PBP) and progressive spinal atrophy (PMA) diseases and used MND as the overall term for the collective cohort. Medical records were reviewed, and subjects for whom the MND diagnosis was uncertain or likely incorrect were excluded. Other exclusion criteria were a diagnosis of spinobulbar muscular atrophy (SBMA) or missing identification data (Swedish social security number) (Fig. 1). A higher percentage of individuals with *C9ORF72HRE* than assumed in an average population with European ancestry with ALS were selected to compare differences in the prevalence of *HTT* expansions and phenotypes between the groups. Almost half of the participants were from the four northernmost counties in Sweden, for which Umeå University Hospital is the tertiary referral hospital (Supplementary Table 1). Healthy relatives (mostly spouses of patients) without a known genetic relationship to an MND patient were selected as controls (Fig. 1). The control group was not age- or sex-matched to the MND cohort but had similar geographical origin (Supplementary Table 1). The study was approved by the Swedish Ethical Review Authority (FEK 94–135, with later updates, most recently EPN 2018–496-32 M for DNA analysis, EPN 2014–17-31 M for the autopsy studies). Written informed consent, including permission to publish the results, was obtained from all participants. The study was performed adhering to the 1964 Declaration of Helsinki (WMA) with later amendments.

### Data collection

Clinical characteristics were collected from medical records (summarized in Table 2). For individuals with IA or RP expansions in *HTT*, medical records were reviewed to collect additional information concerning a history of changes in mood or personality, anxiety, extrapyramidal symptoms, or cognitive decline. Peripheral blood was collected by standard venipuncture into EDTA-containing tubes. After centrifugation, the plasma, buffy coat and erythrocytes were pipetted into separate tubes and stored at −80°C until analysis. Genomic DNA was extracted using NUCLEON BACC3 (Cytiva, Global Life Sciences Solutions, Buckinghamshire, UK) according to the manufacturer’s recommendations. Autopsies were performed on three patients with IA and RP *HTT* expansions, all within 48 h postmortem. CNS and peripheral tissue samples were either fixed in formalin and subsequently embedded in paraffin for immunohistochemical analyses or immediately frozen and stored at −80°C for subsequent DNA preparation [37]. Supplementary Table 2 lists the studied tissue specimens that were collected postmortem.

### DNA and repeat expansion analysis

Venous peripheral blood was collected and DNA extracted from blood leucocytes as described [38]. Patient samples were screened for a panel of ALS genes as described [39, 40]. Analysis of the GGGGCC repeat expansion in intron 1a of C*9ORF72* was performed using repeat-primed PCR (RP-PCR) and fragment length analysis as previously described [38]. In some cases, confirmatory Southern blotting was also performed [38]. A pathological expansion was defined as > 30 repeats in *C9ORF72,* and sequences with < 20 repeats were categorized as not expanded [41, 42]. Intermediate repeat expansions (20–30 repeats) were excluded (20 samples) since it is unclear whether repeat sizes in this range affect the risk of ALS and/or the phenotype [43]. The *HTT* expansion size was identified using a modified version of RP-PCR (described in [8]); primers and cycling conditions were the same as previously described, but an Accuprime reaction mixture was used (Invitrogen), and Q-solution (Qiagen) was added. *HTT* fragment length was identified through fragment PCR using OneTaq master mix, BioLabs and HD primers (for PCR conditions, see Supplementary Table 3 and primer sequences in Supplementary Table 4). The samples were analyzed using a 3500XL Genetic Analyzer (Applied Biosystems, Life Technologies, Singapore) and Peak Scanner version 3.0 software. For *HTT,* a repeat size of 27–35 was categorized as intermediate range, and a repeat size of 36–39 was categorized as reduced penetrance, as proposed by ref. (24). However, no further sequencing to analyze the loss of Cytosine-Adenine-Adenine interruption in *HTT* has been performed.

### Immunohistochemistry analysis

Tissues were collected postmortem from the cervical, thoracic and lumbar regions of the spinal cord as well as from the brain, brainstem and cerebellum, and tissues were dissected for further histological processing (Supplementary Table 2). All the tissues were immersed and fixed in 4% paraformaldehyde in 0.1 M Na phosphate, pH 7.4, at room temperature. Paraffin-embedded sections (4 μm) were stained with hematoxylin and eosin. Immunohistochemical staining was performed with antibodies against HTT, p62, pTDP-43, Tau (AT8), β-amyloid and α-synuclein according to the manufacturer’s instructions using the ES system and ES reagents (Ventana Medical Systems Inc., Illkirch-Graffenstaden, France). A complete list of the commercially available antibodies that were used is listed in Supplementary Table 5. Biotin-conjugated secondary antibodies coupled to an avidin-horseradish peroxidase conjugate 3-amino-9-ethylcarbazole (brown color) were used. Sections were counterstained with hematoxylin, washed, and mounted with Glycergel Mounting Medium (DakoCytomation). Neuropathological assessment was performed by two independent raters (E.S. and K.F.), blinded to the number of *HTT* CAG repeats. Comparisons were made with CNS tissue from a patient with HD, three patients with ALS without *HTT* expansion, three patients with Parkinson’s disease and four individuals without a neurodegenerative disease.

### Statistical analysis

Fisher’s exact test was used to evaluate the association between *HTT* gene expansion and the two MND groups and controls and to evaluate the association between *HTT* IA gene expansion and the MND phenotype. All p values were calculated using R studio software (version 2023.12.0 + 369) with the stats 4.3.2 package. A p value less than 0.05 was considered to indicate a significant difference. Due to the small sample size, no statistical calculations were performed for individuals with full penetrance *HTT* mutations or individuals with respiratory phenotype.

## Supplementary Material

Supplementary_ddae137

## Data Availability

Data is available upon reasonable request.
