# Supplementary material for: Small striatal huntingtin inclusions in patients with motor neuron disease with reduced penetrance and intermediate HTT gene expansions
Source: Hum Mol Genet. 2024 Sep 13;33(22):1966–74. doi: 10.1093/hmg/ddae137 (PMC11555821; doi:10.1093/hmg/ddae137)
Supplement: Supplementary_ddae137 [file supplementary_ddae137.zip › Supplementary_ddae137/Supplementary_Table_S1.docx]

| **Place of birth** | **MND with C9ORF72HRE**  **(302)** | **MND without C9ORF72HRE**  **(514)** | **Control group**  **(352)** |
| --- | --- | --- | --- |
| Born in the north of Sweden (Norrland) | 151 (50%) | 213 (41.4%) | 184 (52.3%) |
| Born in other parts of Sweden | 150 (49.7%) | 298 (58%) | 149 (42.3%) |
| Born in other countries | 0 | 1 (0.2%) | 12 (3.4%) |
| Place of birth unknown | 1 (0.3%) | 2 (0.4%) | 7 (2%) |

**Supplementary Table S1. Place of birth for individuals within the MND cohort and control group.**
