# Supplementary material for: Small striatal huntingtin inclusions in patients with motor neuron disease with reduced penetrance and intermediate HTT gene expansions
Source: Hum Mol Genet. 2024 Sep 13;33(22):1966–74. doi: 10.1093/hmg/ddae137 (PMC11555821; doi:10.1093/hmg/ddae137)
Supplement: Supplementary_ddae137 [file supplementary_ddae137.zip › Supplementary_ddae137/Supplementary_Table_S2.docx]

|  | Hematoxylin and eosin  and  immunohistochemistry | Somatic instability |
| --- | --- | --- |
| Frontal lobe medial | X | X |
| Frontal lobe lateral | X |  |
| Parietal lobe lateral | X | X |
| Gyrus precentralis | X |  |
| Temporal lobe | X | X |
| Occipital lobe | X |  |
| Striatum (Nc. caudatus) | X | X |
| Basal ganglia | X | X |
| Hippocampus | X | X |
| Thalamus | X |  |
| Mesencephalon | X | X |
| Pons | X |  |
| Medulla oblongata | X |  |
| Cerebellum | X | X |
| Spinal cord cervical | X | X |
| Spinal cord thoracic | X | X |
| Spinal cord lumbal | X | X |
| Musculus temporalis |  | X |
| Musculus psoas major |  | X |
| Liver |  | X |
| Heart |  | X |
| Skin |  | X |
| Testis |  | (X, only patient #2) |

**Supplementary Table S2. Description of post-mortem tissues**
