# Supplementary material for: Small striatal huntingtin inclusions in patients with motor neuron disease with reduced penetrance and intermediate HTT gene expansions
Source: Hum Mol Genet. 2024 Sep 13;33(22):1966–74. doi: 10.1093/hmg/ddae137 (PMC11555821; doi:10.1093/hmg/ddae137)
Supplement: Supplementary_ddae137 [file supplementary_ddae137.zip › Supplementary_ddae137/Supplementary_Table_S3.docx]

| 98˚C | 5 min |
| --- | --- |
| 98 ˚C | 45 sec |
| 63˚C | 60 sec 30 cycles |
| 72˚C | 90 sec |
| 72˚C | 10 min |
| 10˚C | ∞ |

**Supplementary Table S3. PCR conditions for HTT fragment analysis PCR.**

| 98˚C | 5 min |
| --- | --- |
| 98 ˚C | 45 sec |
| 63˚C | 60 sec 30 cycles |
| 72˚C | 90 sec |
| 72˚C | 10 min |
| 10˚C | ∞ |

| 98˚C | 5 min |
| --- | --- |
| 98 ˚C | 45 sec |
| 63˚C | 60 sec 30 cycles |
| 72˚C | 90 sec |
| 72˚C | 10 min |
| 10˚C | ∞ |
