# Supplementary material for: Small striatal huntingtin inclusions in patients with motor neuron disease with reduced penetrance and intermediate HTT gene expansions
Source: Hum Mol Genet. 2024 Sep 13;33(22):1966–74. doi: 10.1093/hmg/ddae137 (PMC11555821; doi:10.1093/hmg/ddae137)
Supplement: Supplementary_ddae137 [file supplementary_ddae137.zip › Supplementary_ddae137/Supplementary_Table_S4.docx]

| **PCR primers** | |
| --- | --- |
| **C9orf72** | C9orf72-Fw: 5'-TACGCATCCCAGTTTGAGACGGGGGCCGGGGCCGGGGCCGGGG-3' |
|  | C9orf72-Rev FAM: 5'-AGTCGCTAGAGGCGAAAGC-3' |
|  | C9orf72-Anchor: 5'-TACGCATCCCAGTTTGAGACG-3' |
| **Fragment length primers** | |
| **C9orf72** | C9orf72-Fragment-Rev: 5'-GCAGGCACCCGAACCGCAG-3' |
|  | C9orf72-Fragment-Fw FAM 5'-CAAGGAGGGAAACAACCGCAGCC-3' |
| **HTT** | HD-Fw: 5'-CCT TCG AGT CCC TCA AGT CCT T-3' |
|  | HD-Rev FAM: 5'- GTG GCG GCT GTT G-3' |
| **ATXN-2** | SCA2-Fw: 5'- GGG CCC CTC ACC ATG TCG -3' |
|  | SCA2-Rev FAM: 5'- CGG GCT TGC GGA CAT TGG -3' |

**Supplementary Table S4: PCR primers and primers used for identification of fragment length.**
