# Supplementary material for: Small striatal huntingtin inclusions in patients with motor neuron disease with reduced penetrance and intermediate HTT gene expansions
Source: Hum Mol Genet. 2024 Sep 13;33(22):1966–74. doi: 10.1093/hmg/ddae137 (PMC11555821; doi:10.1093/hmg/ddae137)
Supplement: Supplementary_ddae137 [file supplementary_ddae137.zip › Supplementary_ddae137/Supplementary_Table_S5.docx]

|  | | | |
| --- | --- | --- | --- |
| Description | Product identification | Manufacturer | Antibody dilution |
| Mouse anti-HTT | MAB5374 | Chemicon | 1:50 |
| Mouse anti-HTT | MAB5492 | Chemicon | 1:500 |
| Mouse anti-PEDM | MAB1574 | Chemicon | 1:450 |
| Mouse anti-p62 | 610833 | BD Transduction Laboratories | 1:50 |
| Mouse anti-TDP-43 | CAC-TIP-PTD-M01A | Cosmo Bio LTD | 1:3000 |
| Mouse anti-human Beta-amyloid | M087201-2 | Agilent Technologies | 1:100 |
| Mouse- α-Synuclein | NCL-L-ASYN | Novacastra | 1:100 |
| Mouse anti-TAU(AT8) | BR-03 | Immunogenetics | 1:800 |
| Rabbit anti-SOD1 | SOD1 aa131-153 antibody | In house | 1:800 |

**Supplementary Table S5: Antibodies used in the current study.**
