# Supplementary material for: Small striatal huntingtin inclusions in patients with motor neuron disease with reduced penetrance and intermediate HTT gene expansions
Source: Hum Mol Genet. 2024 Sep 13;33(22):1966–74. doi: 10.1093/hmg/ddae137 (PMC11555821; doi:10.1093/hmg/ddae137)
Supplement: Supplementary_ddae137 [file supplementary_ddae137.zip › Supplementary_ddae137/Supplementary_Table_S6.docx]

|  | *HTT-*status | Other gene expansions | Diagnosis | Behavioural/cognitive/ extrapyramidal symptoms | First symptom | Age of onset (years) | Disease duration (months) | Sex |
| --- | --- | --- | --- | --- | --- | --- | --- | --- |
| 1 | 35-9 | *C9ORF72HRE* | ALS | N/A | hand | 61 | 32 | F |
| 2 | 33-19 | - | ALS | N/A | leg | 52 | 73 | F |
| 3 | 33-16 | - | ALS | N/A | leg | 49 | 128 | F |
| 4 | 33-22 | - | PMA | Anxiety. Depression. Short time memory influenced. No information of other motoric symptoms. | arm | 67 | 62 | F |
| 5 | 33-15 | *C9ORF72HRE* | ALS | No documented symptoms | arm, leg | 55 | 32 | M |
| 6 | 33-22 | *C9ORF72HRE* | ALS | No cognitive, behavioural or extrapyramidal symptoms | hand | 52 | 41 | M |
| 7 | 32-17 | - | ALS | Heredity. Difficulties with concentration and information procession. Reduced strenght, fine motor skills, spasticity. | arm | 22 | Alive, after 9 years | F |
| 8 | 32-18 | *ATXN2*  *(33-23)* | PBP | No cognitive or emotional, no extrapyramidal symptoms | dysartria | 85 | alive after 4 years 8 months | F |
| 9 | 32-20 | *C9ORF72HRE* | ALS | N/A | hand | 57 | 18 | M |
| 10 | 32-17 | - | ALS | Emotional instability, episode with depression.  Grandmother with PD, son with OCD. | dysarthria | 44 | alive after 3 years, 7 months | F |
| 11 | 32-21 | *C9ORF72HRE* | ALS | N/A |  |  |  | M |
| 12 | 31-18 | - | PBP | N/A | bulbar | 69 | 19 | F |
| 13 | 30-17 | - | ALS | No cognitive, behavioural or extrapyramidal symptoms. | dysphonia, dysphagia | 67 | 53 | M |
| 14 | 30-25 | - | ALS | N/A | leg | 77 | 21 | M |
| 15 | 30-24 | - | PBP-aphasia | N/A | aphasia | 65 | 25 | F |
| 16 | 30-17 | *C9ORF72HRE* | ALS | No cognitive, behavioural, no extrapyramidal symptoms | arm | 48 | 16 | F |
| 17 | 30-21 | *C9ORF72HRE* | PBP | N/A | bulbar | 63 | 70 | F |
| 18 | 30-17 | *C9ORF72HRE* | PBP | N/A | dysarthria | 60 | 26 | F |
| 19 | 30-X | - | PMA | N/A | spinal | 46 | 69 | F |
| 20 | 29-18 | - | ALS | No cognitive or behavioural, no extrapyramidal symptoms | arm and leg | 61 | Alive, after 8 years | F |
| 21 | 29-18 | - | ALS | N/A | leg | 71 | 31 | F |
| 22 | 29-17 | - | ALS | N/A | arm | 75 | 36 | F |
| 23 | 29-17 | - | ALS | No cognitive or behavioural, no extrapyramidal symptoms | leg stiffness | 22 | alive after 19,5 years | F |
| 24 | 29-18 | - | MND | N/A |  |  |  | F |
| 25 | 29-22 | *C9ORF72HRE* | PBP | N/A | bulbar | 52 | 17 | M |
| 26 | 29-18 | *C9ORF72HRE* | PBP | N/A | bulbar | 50 | 56 | M |
| 27 | 28-17 | (not from Sweden) | ALS | No cognitive, behavioural or extra pyramidal symptoms | hand | 39 | 40 | M |
| 28 | 28-22 | - | ALS | N/A | leg | 81 | 22 | F |
| 29 | 28-20 | - | ALS | No cognitive, behavioural or extrapyramidal symptoms | hand | 54 | Alive after 3 years, 3 months | M |
| 30 | 28-9 | - | PBP | Mother with ALS.  Depression, anxiety 20 years before disease, no behavioural or cognitive symptoms | dysartri | 67 | Alive after 2 years 11 months | F |
| 31 | 28-19 | - | PMA | N/A | leg | 70 | 43 | M |
| 32 | 28-15 | - | ALS | N/A | hand | 41 | 43 | M |
| 33 | 27-20 | - | ALS | No cognitive, behavioural or extrapyramidal symptoms | leg | 56 | Alive, after 4 years 10 months | F |
| 34 | 27-18 | - | ALS | N/A | leg | 62 | 21 | M |
| 35 | 27-21 | - | ALS | No cognitive, behavioural or extrapyramidal. | leg | 39 | Alive,13 years later | M |
| 36 | 27-23 | - | ALS | N/A | arm | 76 | 22 | M |
| 37 | 27-17 | - | ALS | No cognitive, behavioural or extra pyramidal symptoms | arm | 49 | 39 | M |
| 38 | 27-17 | - | ALS | No cognitive, behavioural or extrapyramidal symptoms | arm | 33 | Alive, after 20 years | F |
| 39 | 27-17 | - | MND | N/A | leg | 64 | 20 | M |
| 40 | 27-18 | - | PBP | No behavioural, cognitive or extrapyramidal symptoms | dysartria | 70 | Alive, after 4 years 1 month | F |
| 41 | 27-17 | - | PBP | N/A | bulbar | 56 | 35 | F |
| 42 | 27-17 | - | PBP | N/A | bulbar | 70 | 13 | F |
| 43 | 27-17 | - | ALS | No behavioural, cognitive or extrapyramidal symptoms | leg | 60 | 39 | M |
| 44 | 27-18 | *C9ORF72HRE* | ALS | Mother with FTD. No psychiatric or extra pyramidal symptoms. Cognitive symptoms: memory and concentration. ECAS: described as slightly reduced. | leg | 64 | 58 | M |
| 45 | 27-17 | *C9ORF72HRE* | ALS | N/A |  |  |  | F |
| 46 | 27-18 | *C9ORF72HRE* | ALS | N/A | leg | 49 | 60 | F |
| 47 | 27-17 | *C9ORF72HRE* | ALS | N/A | arm/respiratory | 59 | 31 | M |
| 48 | 27-17 | *C9ORF72HRE* | ALS | N/A | hand | 52 | 55 | F |
| 49 | 27-18 | - | PMA | No cognitive or behavioral symptoms, tremor in hands, no other extrapyramidal symptoms. | leg | 67 | 60 | M |
| 50 | 27-19 | *C9ORF72HRE*  *ATXN2*  *(29-22)* | MND-FTD | N/A | personality | 59 | 40 | M |
| 51 | 27-27 | *C9ORF72HRE* | PMA | N/A | arm | 62 | 33 | M |

**Supplementary Table S6. Clinical data for individuals with MND and HTT intermediate repeat expansions.**

N/ A: information not available. ALS, amyotrophic lateral sclerosis; FTD, frontotemporal lobe dementia; MND, motor neuron disease; PBP, progressive bulbar paresis.

PMA, progressive muscle atrophy; C9ORF72HRE, hexanucleotide repeat expansion in C9ORF72; HTT, gene associated with Huntington´s disease; ATXN2, gene associated with spinocerebellar ataxia 2.
