# Supplementary material for: Small striatal huntingtin inclusions in patients with motor neuron disease with reduced penetrance and intermediate HTT gene expansions
Source: Hum Mol Genet. 2024 Sep 13;33(22):1966–74. doi: 10.1093/hmg/ddae137 (PMC11555821; doi:10.1093/hmg/ddae137)
Supplement: Supplementary_ddae137 [file supplementary_ddae137.zip › Supplementary_ddae137/Supplementary_Table_S7.docx]

| **Patient number** | ***1*** | **2** | **3** | **4** | **5** |
| --- | --- | --- | --- | --- | --- |
| Sex | Female | Male | Male | Male | Female |
| Age at onset (years) | 63 | 63 | 71 | 83 | 51 |
| Age at death (years) | 66 | 68 | 75 | 85 | 54 |
| Disease duration (months) | *31* | 61 | 46 | 15 | 40 |
| First symptom | dysarthria, bulbar | Parkinsonism,  peroneus paresis | hand | dysarthria, dysphagia | foot |
| Behavioural/cognitive/extrapyramidal signs | Not described | Bipolar disease, anxiety. Postural tremor. Gait disturbance with shortened stride length, extra steps when turning. Hypokinesia. Impaired fine motor skills. Unstable mood, no affect lability. | ECAS 88/136  Tremor, impaired fine motor skills, stiffness, dystonia. | Short-term memory affected, irritability. | Not described |
| *HTT*-status | 38-20 | 37-20 | 36-19 | 36-17 | 36-21 |
| Concomittant gene expansions | *C9ORF72* | *ATXN2: 29-22* | - | - | *C9ORF72* |
| Diagnosis | PBP | ALS | ALS | PBP | ALS |

**Supplementary Table S7. Clinical data for individuals with MND and HTT reduced penetrance gene expansion.**

ALS, amyotrophic lateral sclerosis; ATXN2, gene associated with spinocerebellar ataxia 2; C9ORF72HRE, hexanucleotide repeat expansion in C9ORF72; ECAS, Edinburgh Cognitive and Behavioural ALS screen; HTT, gene associated with Huntington´s disease; MND, motor neuron disease; PBP, progressive bulbar paresis.
