# Supplementary material for: Small striatal huntingtin inclusions in patients with motor neuron disease with reduced penetrance and intermediate HTT gene expansions
Source: Hum Mol Genet. 2024 Sep 13;33(22):1966–74. doi: 10.1093/hmg/ddae137 (PMC11555821; doi:10.1093/hmg/ddae137)
Supplement: Supplementary_ddae137 [file supplementary_ddae137.zip › Supplementary_ddae137/Supplementary_Table_S8.docx]

| **Patient number** | ***1*** | **2** | **3** |
| --- | --- | --- | --- |
| Sex | Male | Male | Woman |
| Age at onset (years) | 71 | 83 | 67 |
| Age at death (years) | 75 | 85 | 72 |
| Disease duration (months) | 46 | 15 | 62 |
| First symptom | Weakness left hand and arm | dysarthria, dysphagia | Weakness right arm |
| Behavioural/cognitive/extrapyramidal signs | ECAS 88/136: pathological results on executive function, frontal lobe specific tests.  Tremor in hands, impaired fine motor skills, stiffness in hands, neck and body, dystonia. Anxiety, no depression. Twitches in face. No chorea. | Short-term memory affected, irritability. | Complains about short time memory and anxiety. Listlessness and dejection. No chorea. Balance deteriorates. No extrapyramidal symptoms. |
| *HTT*-status | 36-19 | 36-17 | 33-22 |
| Concomitant gene expansions (*C9ORF72* or *ATXN2*) | no | no | no |
| Diagnosis | ALS | PBP | PMA |
| Concomitant diseases/treatments | Hypertonia. Radiotherapy due to prostate cancer. | ECRP due to cholecystitis. CT scan of the brain shows right sided ischemia. | Meningitis as a teenager. Smoker. TIA at the age of 53. Peripheral facial paresis at the age of 64. |
| Heredity for dementia or MND | No | No | - |

**Supplementary Table S8. Clinical data for autopsied individuals with MND and HTT intermediate or reduced penetrance expanded alleles.**

ALS, amyotrophic lateral sclerosis; ATXN2, gene associated with spinocerebellar ataxia 2; C9ORF72HRE, hexanucleotide repeat expansion in C9ORF72; ECAS, Edinburgh Cognitive and Behavioural ALS screen; ECRP, endoscopic retrograde cholangiopancreatography; HTT, gene associated with Huntington´s disease; MND, motor neuron disease; PBP, progressive bulbar paresis; PMA, progressive muscle atrophy; TIA, transitory ischemic attack.
