# Supplementary figures and images for: Small striatal huntingtin inclusions in patients with motor neuron disease with reduced penetrance and intermediate HTT gene expansions
Source: Hum Mol Genet. 2024 Sep 13;33(22):1966–74. doi: 10.1093/hmg/ddae137 (PMC11555821; doi:10.1093/hmg/ddae137)

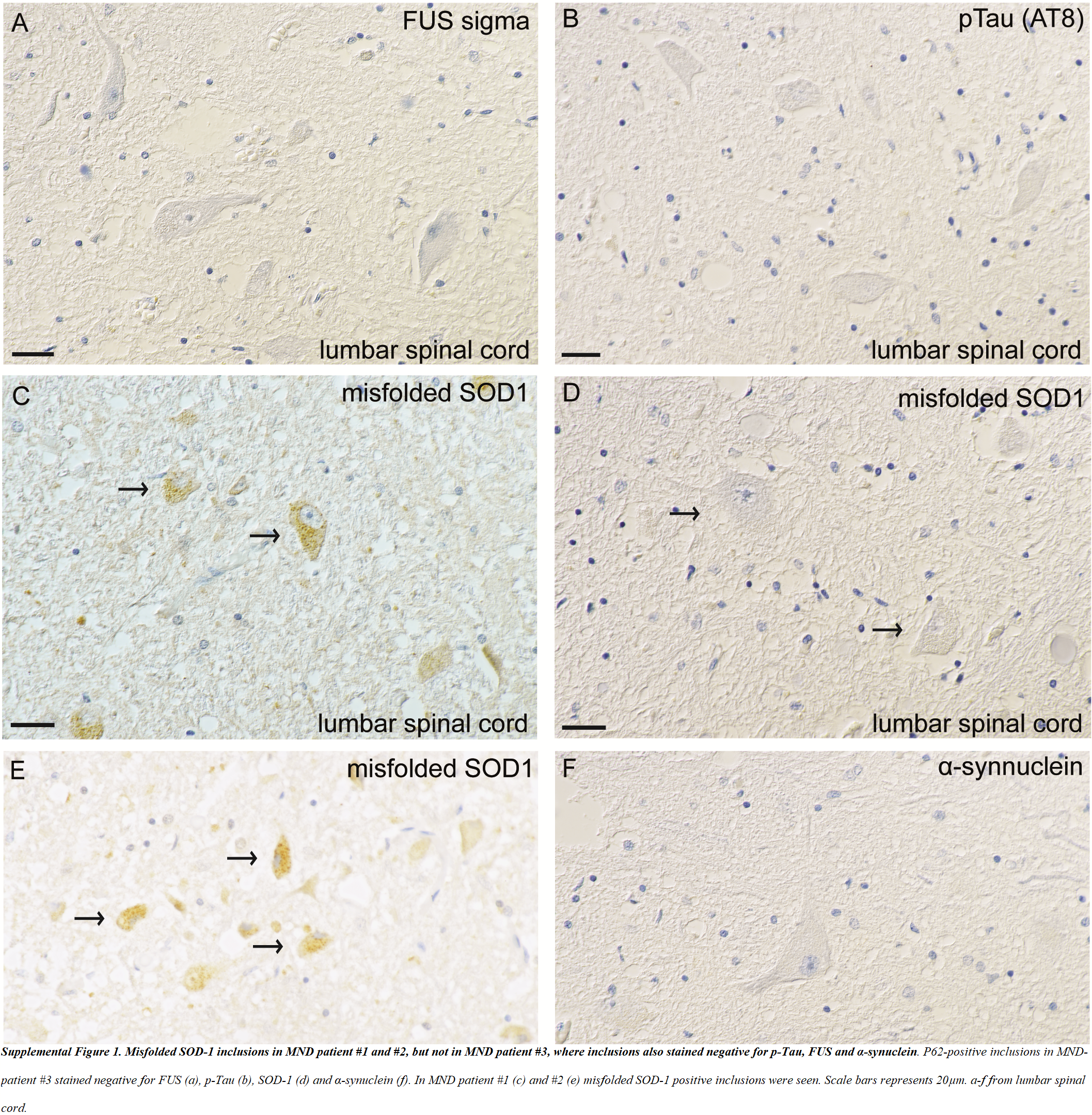

Supplement: Supplementary_ddae137 [file supplementary_ddae137.zip › Supplementary_ddae137/Supplement Fig 1.tif]
